# Gender dimorphism in hepatocarcinogenesis—DNA methylation modification regulated X‐chromosome inactivation escape molecule *XIST*

**DOI:** 10.1002/ctm2.1518

**Published:** 2023-12-26

**Authors:** Zhihui Dai, Sijie Wang, Xinggang Guo, Yuefan Wang, Haozan Yin, Jian Tan, Chenyang Mu, Shu‐Han Sun, Hui Liu, Fu Yang

**Affiliations:** ^1^ Department of Medical Genetics Naval Medical University Shanghai China; ^2^ School of Health Science and Engineering University of Shanghai for Science and Technology Shanghai China; ^3^ Third Department of Hepatic Surgery Eastern Hepatobiliary Surgery Hospital, Naval Medical University Shanghai China; ^4^ Shanghai Key Laboratory of Medical Bioprotection Shanghai China; ^5^ Key Laboratory of Biological Defense, Ministry of Education Shanghai China

**Keywords:** DNA methylation, hepatocellular carcinoma, TET2, XIST

## Abstract

**Background:**

Sex disparities constitute a significant issue in hepatocellular carcinoma (HCC). However, the mechanism of gender dimorphism in HCC is still not completely understood.

**Methods:**

5‐Hydroxymethylcytosine (5hmC)‐Seal technology was utilised to detect the global 5hmC levels from four female and four male HCC samples. Methylation of *XIST* was detected by Sequenom MassARRAY methylation profiling between HCC tissues (T) and adjacent normal liver tissues (L). The role of Tet methylcytosine dioxygenase 2 (TET2) was investigated using diethylnitrosamine (DEN)‐administered *Tet2* ^−/−^ female mice, which regulated *XIST* in hepatocarcinogenesis. All statistical analyses were carried out by GraphPad Prism 9.0 and SPSS version 19.0 software.

**Results:**

The results demonstrated that the numbers of 5hmC reads in the first exon of *XIST* from female HCC tissues (T) were remarkably lower than that in female adjacent normal liver tissues (L). Correspondingly, DNA methylation level of *XIST* first exon region was significantly increased in female T than in L. By contrast, no significant change was observed in male HCC patients. Compared to L, the expression of *XIST* in T was also significantly downregulated. Female patients with higher *XIST* in HCC had a higher overall survival (OS) and more extended recurrence‐free survival (RFS). Moreover, TET2 can interact with YY1 binding to the promoter region of *XIST* and maintain the hypomethylation state of *XIST*. In addition, DEN‐administered *Tet2* ^−/−^ mice developed more tumours than controls in female mice.

**Conclusions:**

Our study provided that YY1 and TET2 could interact to form protein complexes binding to the promoter region of *XIST*, regulating the methylation level of *XIST* and then affecting the expression of *XIST*. This research will provide a new clue for studying sex disparities in hepatocarcinogenesis.

**Highlights:**

1. *XIST* was significantly downregulated in HCC tissues and had gender disparity.
2. Methylation levels in the *XIST* first exon were higher in female HCC tissues, but no significant change in male HCC patients.
3. The TET2–YY1 complex regulate *XIST* expression in female hepatocytes.
4. Other ways regulate *XIST* expression in male hepatocytes.

## Graphical abstract

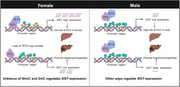

## 1 INTRODUCTION

Primary liver cancer is prevalent and has one of the highest fatality rates, which poses a massive threat to human health.^1^, ^2^ It is more common in men than in women. The incidence rate and mortality of liver cancer in men are 14.1 and 12.9 per 100 000, respectively, while in women, the incidence rate and mortality are 5.2 and 4.8 per 100 000, respectively.^1^ Over the past few decades, researchers believed that sex hormone was the key to the gender differences in hepatocellular carcinoma (HCC). Estrogen inhibits HCC development and procession via miRNAs, DNA repair and obesity‐associated pathways.^3^, ^4^, ^5^, ^6^ Instead, androgen and androgen receptors serve a promoting role.^7^, ^8^ Moreover, the autosome gene *CYP39A1* with female‐preferential expression is a potent inhibitor of HCC development, which provides a possible mechanism for gender differences in HCC.^9^ Meanwhile, some transcripts that escape X chromosome inactivation (XCI) also lead to gender disparity of HCC, such as *FTX*, *JPX* and *XIST*. In HCC, previous studies found that long non‐coding RNA (lncRNA) *FTX* was differentially expressed between sexes, and inhibited HCC proliferation and metastasis.^10^ *JPX* is another non‐coding gene that can activate *XIST*, which balances the activator and inhibitor to control XCI.^11^ Studies also confirmed that *JPX* was decreased in HCC, resulting in inferior overall survival (OS) of HCC.^12^, ^13^ *XIST* is a lncRNA located in the XCI and an essential mediator of X inactivation.^14^ Evidence accumulated suggested that *XIST* played critical regulatory roles in sex disparities diseases, such as autoimmune diseases, sex disparities cancers, neurological disorders and so on.^15^ However, molecular mechanisms still need to be explored in depth.

Earlier studies had found an overall tendency to DNA hypomethylation in many cancers.^16^ Sex‐specific methylation patterns were also extensive in the liver.^17^, ^18^ DNA methylation is a dynamic process accompanied by DNA demethylation. 5‐Methylcytosine (5mC) can be gradually oxidised by Tet methylcytosine dioxygenase (TET) enzymes (TET1, TET2 and TET3) to 5‐hydroxymethylcytosine (5hmC).^19^, ^20^ Due to the wide distribution of 5mC and 5hmC in the human genome, high chemical stability and close correlation with gene expression, some scholars have proposed that 5mC and 5hmC can be applied as an ideal biomarker for cancer diagnosis.^21^

Li et al. collected samples from colorectal cancer (CC), gastric cancer (GC), HCC, thyroid, pancreatic cancer and healthy individuals, analysed 5hmC levels of circulating cell‐free DNA (cfDNA) in plasma and determined 5hmC levels of genomic DNA (gDNA) in tumour tissues and the adjacent healthy tissues. They found that the global 5hmC level of tumour gDNA was significantly downregulated. Compared with control plasma cfDNA, 5hmC levels of cancer patient's cfDNA also had lower levels.^21^ Cai et al. applied 5hmC‐Seal technology to capture 5hmC sequences in cfDNA samples from 2554 Chinese subjects and found that 5hmC can be a marker for HCC staging and prognosis.^22^ Interestingly, the incidence of CC, GC, HCC, thyroid and pancreatic cancer all exist in sex dimorphism.^23^, ^24^ Unfortunately, these studies did not distinguish the gender of tissue samples.

In this study, we first use 5hmC‐Seal technology to detect whether there are gender differences in 5hmC distribution from four female and four male HCC patients. Results demonstrated that 5hmC distribution was significantly different between female and male HCC patients. The number of 5hmC reads in the first exon of *XIST* from female HCC tissues was remarkably lower than in adjacent normal liver tissues. Then, we clarify the regulatory mechanism of *XIST*’s tumour suppressive function on female HCC. Studies may offer a novel understanding of the mechanisms of HCC gender disparity.

## 2 MATERIALS AND METHODS

### 2.1 Patient samples and clinical data

We randomly obtained 112 pairs (cohorts 1, 2 and 3) of HCC and adjacent normal liver tissues (at least 3 cm away from the tumour border and with no microscopic tumour cells) from Eastern Hepatobiliary Hospital (Shanghai, China) operated between 2009 and 2016 (including complete clinical and follow‐up data). The samples were stored at −80°C until experimental treatment performed. The clinical characteristics of patients are listed in Tables S1 and S2. This research was approved by the ethics committee of the Naval Medical University. All patients signed the informed consent.

### 2.2 5hmC‐Seal sequencing

5hmC‐Seal sequencing was performed as described previously.^25^ DNA extraction, DNA quantification and qualification, library preparation and quantification, sequencing and data analysis were all performed by the Yunbios platform (Yunbios).

### 2.3 Cell lines and cell culture

HCC‐1016 and HCC‐3527 (female primary HCC cell lines) were obtained from the tumour tissues of HCC patients.^26^ Cells were cultured in a high‐glucose Dulbecco's Modified Eagle's Medium (DMEM) with 10% foetal bovine serum at 37°C, 5% CO_2_, and kept the humidity at a certain level.

### 2.4 Sequenom MassARRAY methylation

Quantitative methylation analysis of the *XIST* first exon region of HCC patients and the *XIST* exon region of mice was performed by using Sequenom MassARRAY methylation spectroscopy (CapitalBio). The primers used to amplify target regions are listed in Table S3.

### 2.5 hMeDIP‐qPCR and MeDIP‐qPCR assay

We performed immunoprecipitation using the Hydroxymethylated DNA Immunoprecipitation (hMeDIP) kit (ab117134, Abcam) and quantified the enrichment of 5hmC DNA at the target loci by quantitative real‐time polymerase chain reaction (qPCR). Methylated DNA Immunoprecipitation (MeDIP) kit (ab117135, Abcam) was used to quantified the enrichment of 5mC DNA at the target loci according the user manual. The primers are shown in Table S3.

### 2.6 TaqMan copy number assay

We determined the *TET2* gene copy number by TaqMan gene copy number assay in female samples using the RNaseP gene as a standard reference gene according to the instructions. Cycling conditions were: 95°C for 10 min and 40 cycles of 95°C for 15 s and 60°C for 1 min. The copy number of target gene was analysed and calculated by CopyCaller software 2.1 (Applied Biosystems).

### 2.7 Chromatin immunoprecipitation

The SimpleChIP Plus Enzyme Chromatin IP Kit (#9005, CST) was used for chromatin immunoprecipitation (ChIP) assays. We incubated for 20 min at room temperature for cross‐linking of the liver tissue mixture, made from mix of finely minced human liver tissue (10 mg) and 42.5 μL of 37% formaldehyde. We homogenise suspended tissue, washed twice with ice‐cold phosphate‐buffered saline (PBS) using a B‐type Dounce homogeniser, suspended by centrifugation after adding glycine to stop the cross‐linking reaction. Then, resuspended in Kit Buffer A and incubated with micrococcal nuclease for 20 min at 37°C. We disrupted nuclei by sonication, removed debris by centrifugation and treated clarified nuclear extracts with TET2 antibody (ab94580, Abcam, 1:50) or YY1 antibody (ab109228, Abcam, 1:100). Immunoprecipitation with protein G magnetic beads was performed after incubation at 4°C overnight. ChIP‐enriched DNA was analysed by qPCR using specific primers, as described in Table S3.

### 2.8 Co‐immunoprecipitation

We performed co‐immunoprecipitation (Co‐IP) using the Thermo Scientific Pierce Co‐IP kit. First, immobilised TET2 antibody (ab94580, Abcam, 1:50) with AminoLink Plus conjugated resin for 2 h, rinsed the resin and incubated it with tissue lysates overnight. Then, re‐washed the resin and eluted the protein using elution buffer using a primary YY1 antibody (ab109228, Abcam, 1:1000) and 800‐CW goat anti‐rabbit immunoglobulin G (IgG) as a secondary antibody (1:5000; LI‐COR Biosciences Inc.) Finally, membranes were analysed using the Odyssey infrared scanner (LI‐COR Biosciences Inc.).

### 2.9 Immunofluorescence staining

We washed HCC‐1016 and HCC‐3527 cells (cultured on 13 mm round glass coverslips) three times with cold PBS. Coverslips with samples were fixed in 3% paraformaldehyde for 30 min and then permeabilised with permeabilisation solution for an additional 30 min. TET antibody (ab94580, Abcam, 1:200) and YY1 antibody (ab199815, Abcam, 1:100) were incubated with cells at room temperature for 1 h. Subsequently, cells were washed three times and incubated with goat anti‐rabbit IgG H&L (ab150077, Abcam) for 1 h. Coverslips were washed five times and then stained with Hoechst 33342 (1 mg/mL; Sigma) for 10 min. Zeiss laser confocal scanning microscope (Carl Zeiss) used to analyse cell samples.

### 2.10 Dual‐luciferase reporter assay

The dual‐luciferase reporter plasmid was purchased from Obio Technology (Shanghai) Corp., Ltd. YY1 binding sites on the *XIST* promoter was analysed by JASPAR database. The luciferase reporter plasmid construct contained the *XIST* promoter containing YY1 binding sites (wild type, WT) or lacking YY1 binding sites (mutant type, MUT). Dual‐luciferase reporter assay was performed using Dual‐Luciferase Reporter Assay Kit (Hanbio) accordance with the manufacturer's instructions. The sequence of *XIST* promoter is listed in Supporting Information.

### 2.11 Animal models

Shanghai Biomodel Organism Science & Technology Development Co., Ltd. was entrusted to knock out the *Tet2* gene of C57BL/6J mice (*Tet2* ^−/−^). Untreated age and sex‐matched littermate C57BL/6J mice were used as corresponding WT mice. All mice were fed freely standard diet and water. Maintain a 12‐h on and 12‐h off light cycle at 24°C ± 2°C and 65% ± 5% humidity. Animal studies were approved by the Institutional Animal Care and Use Committee of Naval Medical University (Shanghai, China) and performed under the National Institutes of Health Guide for the Care and Use of Laboratory Animals. Mouse models of HCC (WT and *Tet2* ^−/−^ mice) were established by intraperitoneally injecting 30 mg/kg body weight of diethylnitrosamine (DEN; Sigma) at 15 days of age. Mice were humanely sacrificed 40 weeks after sample injection. Additional materials and methods are listed in Supporting Information.

### 2.12 Statistical analysis

Student's *t*‐test was used to analysis the difference between two sets of data. A chi‐squared test was applied to verify the relationship between *XIST* and clinicopathological features. For assessing the difference in patient survival, Kaplan–Meier analysis and log‐rank test were used. To identify independent influencing factors based on variables selected by Cox univariate analysis, the Cox proportional hazards model was used. Pearson or Spearman correlation coefficient was used to verify statistical correlation. According to different usage scenarios, the data are statistically significant in the following *p*‐values: ^*^ *p* < .05, ^**^ *p* < .01, ^***^ *p* < .001 and ^****^ *p* < .0001. All statistical analyses were carried out by GraphPad Prism 9.0 and SPSS version 19.0 software.

## 3 RESULTS

### 3.1 5hmC profiles differ between female and male HCC patients

**FIGURE 1:**
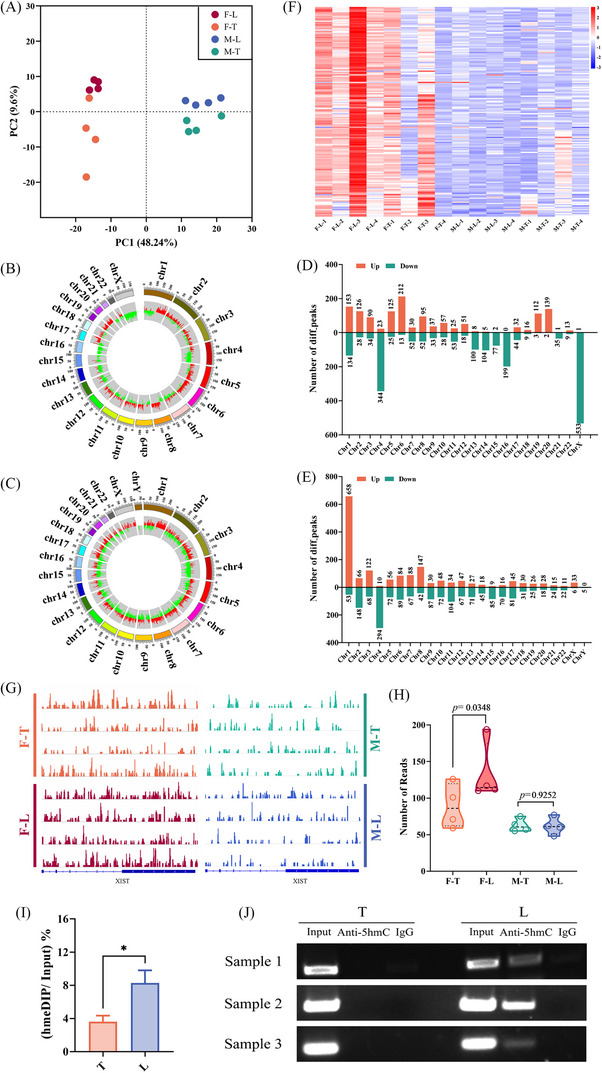
Characteristics of 5‐hydroxymethylcytosine (5hmC) distribution in four female and four male hepatocellular carcinoma (HCC) patients. (A) Principal component analysis (PCA) of genome‐wide hydroxymethylation level in HCC patients. Each data point represents an individual sample. The distribution of the differentia peaks between tumour tissues and adjacent normal liver tissues in female patients (B) and male patients (C) is shown across the human chromosomes. Peaks coloured in red represent significant upregulated hydroxymethylation levels in tumour tissues compared to adjacent normal liver tissues. Peaks coloured in green represent downregulated hydroxymethylation levels in tumour tissues compared to adjacent normal liver tissues. The number of differential peaks between tumour tissues and adjacent normal liver tissues in female patients (D) and male patients (E) located on human chromosomes. (F) Heatmap of 168 genes escaping X chromosome inactivation (XCI) between female and male tissue samples. (G) Integrative Genomics Viewer (IGV) snapshot of *XIST* shown the 5hmC signal peaks in female and male samples. (H) Normalised counts of 5hmC reads in the first exon of *XIST*. (I) The 5hmC level in the *XIST* first exon from three HCC female patients by hMeDIP assay and qPCR. (J) *XIST* was quantified using DNA agarose gel electrophoresis.

5hmC‐Seal sequencing was utilised to generate genome‐wide 5hmC profiles from four female and four male HCC patients. Principal component analysis verified that there was a markedly difference between HCC tissues (T) and adjacent normal liver tissues (L) whether in female or male patients (Figure 1A). In addition, a striking separation can be seen between female and male patients (Figure 1A). Then, we compared the distribution of differential peaks across chromosomes. Overall, the significant difference can be observed across the X chromosome was that there were almost all 5hmC downregulated peaks in female HCC tissues (F‐T) compared to adjacent normal liver tissues (F‐L) (Figure 1B). However, there were almost all upregulated peaks located on the X chromosome in male HCC tissues (M‐T) compared to adjacent normal liver tissues (M‐L) (Figure 1C). Next, we counted the number of different peaks located on human chromosomes. A total of 533 5hmC downregulated peaks and one upregulated peak were located on the X chromosome in females (Figure 1D), but only six downregulated peaks and 33 upregulated peaks were located on the X chromosome in males (Figure 1E). So, there was also a marked difference in the number of different peaks located on the X chromosome between female and male patients.

Given that XCI produce dosage compensation by randomly inactivating one of the X chromosomes in females.^27^ Some genes can escape XCI to protect females from complete functional loss.^28^ The 5hmC reads number of genes (Table S4) escaping XCI on the basis of previous research^29^ was analysed. The heatmap result demonstrated that 5hmC modification signal of the XCI escape gene coding region in M‐T and M‐L was very low, and there was no difference. In contrast, the 5hmC modification signal of the XCI escape gene coding region in F‐T was lower than that in F‐L (Figure 1F).

In addition, *XIST* is located in the XCI and plays a vital role in random XCI.^15^, ^30^ The 5hmC signal peaks in *XIST* were markedly increased in female samples than in male samples (Figure 1G). Further analysis showed that the number of 5hmC reads in the first exon of *XIST* from F‐T were remarkably lower than that in F‐L. However, there was no difference between M‐T and M‐L (Figure 1H). Then, hMeDIP was used to detect 5hmC (hydroxymethylation marker) levels in the first exon of *XIST* in the female HCC patients. The results revealed that the 5hmC level of the *XIST* first exon in F‐T was lower compared to that in F‐L (Figure 1I,J).

### 3.2 Methylation levels in the *XIST* first exon were higher in female HCC tissues

**FIGURE 2:**
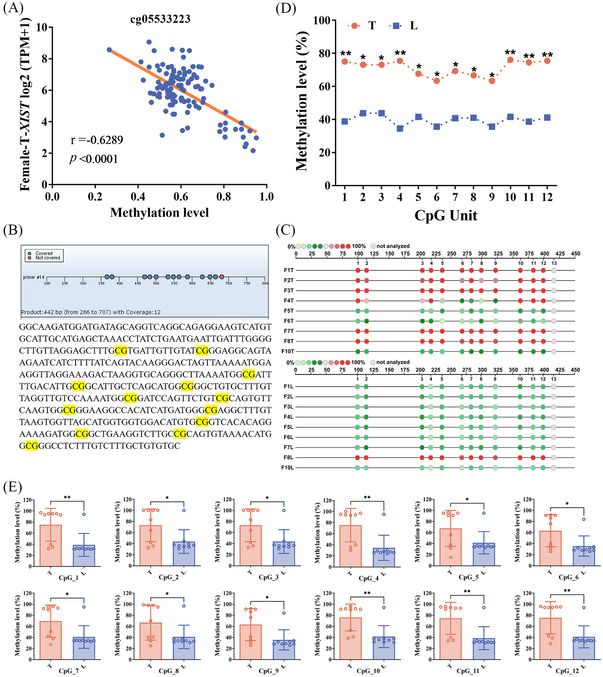
Methylation levels in the *XIST* first exon were higher in female hepatocellular carcinoma (HCC) tissues. (A) Correlation between the level of methylation and *XIST* in 118 female HCC tissues. Pearson correlation analysis was used to measure the correlation of female data. (B) Methylation mass spectrum primers and sequence of *XIST* first exon region (yellow indicates the detection sites, a total of 12). (C) Methylation levels of CpG sites in *XIST* first exon region from nine female patients. Quantitative methylation analysis results are shown in a colour scale: light green (0% methylation), green (50% methylation) and dark red (100% methylation). The grey circles represent the missing data at a given CpG site. (D) Mean methylation levels of CpG sites in *XIST* first exon region from nine pairs of female tumour tissues and adjacent normal liver tissues. (E) Methylation level of each CpG site between the two groups in *XIST* first exon.

**FIGURE 3:**
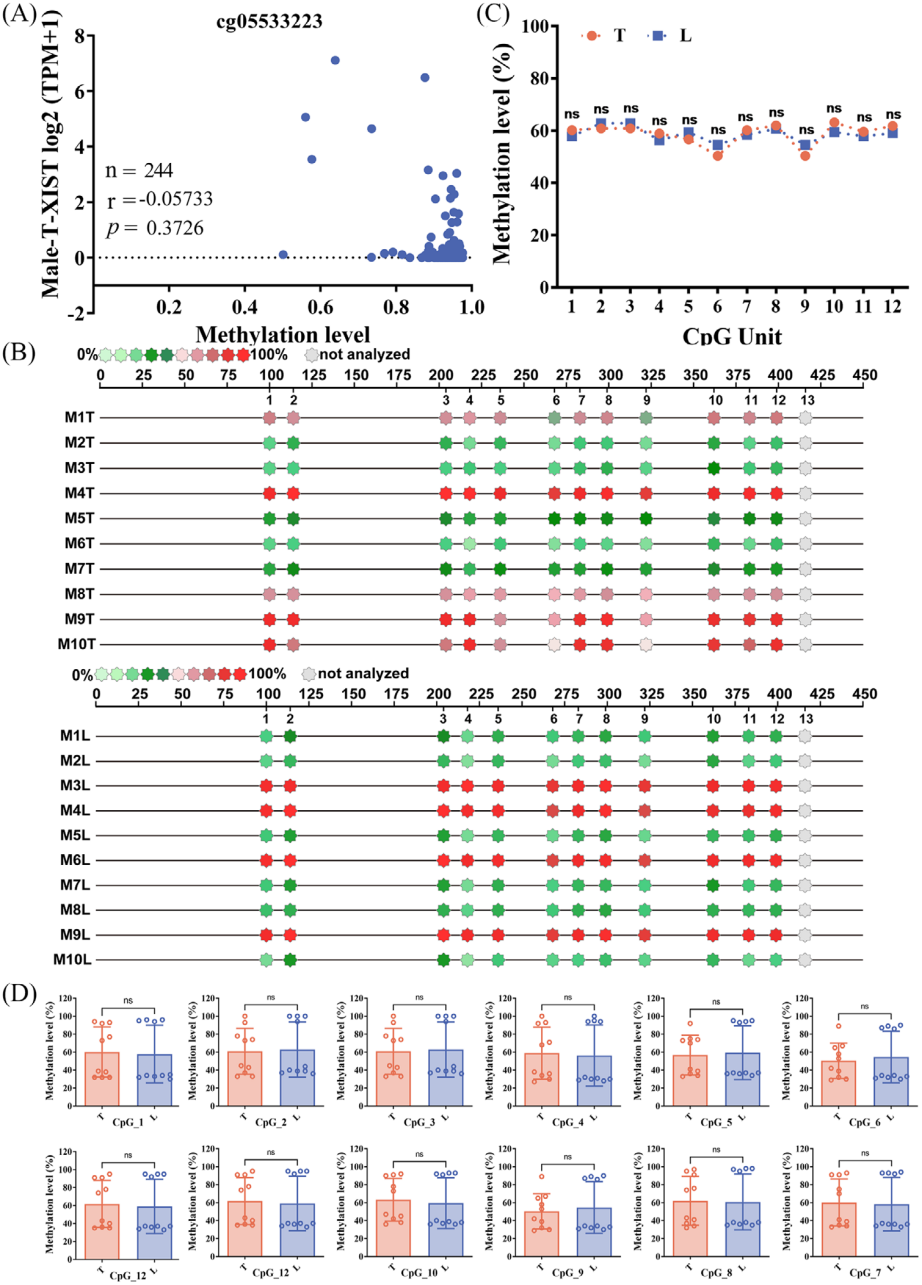
Methylation levels in the *XIST* first exon were no significant change in male hepatocellular carcinoma (HCC) tissues. (A) Correlation between the level of methylation and *XIST* in 244 male HCC tissues. Spearman correlation analysis was used to measure the correlation of female data. (B) Methylation levels of CpG sites in *XIST* first exon region from 10 male patients. Quantitative methylation analysis results are shown in a colour scale: light green (0% methylation), green (50% methylation) and dark red (100% methylation). The grey circles represent the missing data at a given CpG site. (C) Mean methylation levels of CpG sites in *XIST* first exon region in 10 pairs of male tumour tissues and adjacent normal liver tissues. (D) Methylation level of each CpG site between the two groups in the *XIST* first exon.

Subsequently, we examined the correlation between the level of DNA methylation and *XIST* using the Shiny Methylation Analysis Resource Tool (SMART, http://www.bioinfo‐zs.com/smartapp/) in HCC samples. We select the probe (cg:05533223, cg:03554089 and cg:12653510) located in *XIST* first exon region for data analysis. An extremely negative correlation was seen between *XIST* expression and methylation in female HCC tissues (Figures 2A and S1A,B). In male HCC tissues, a significantly negative correlation was observed between *XIST* expression and methylation at the probe cg:12653510 (Figure S1C). However, there was no correlation at the other two probes (Figures 3A and S1D) in male HCC tissues.

Then, the methylation level of *XIST* first exon region was detected by Sequenom MassARRAY methylation profiling. The detection primers (the probe cg05533223 was also located in the target sequence, Figure 2B) for *XIST* first exon region were designed to detect nine female HCC patients and 10 male HCC patients. Twelve Cytosine Guanine dinucleotide (CpG) sites were analysed in this region. We quantified the level of CpG site methylation in *XIST* first exon region and compared between T and L. For female patients, methylation levels at all CpG sites in T were conspicuously higher than in L (Figure 2C–E). Conversely, no significant change was observed in male HCC patients (Figure 3B–D).

### 3.3 *XIST* was significantly downregulated in HCC tissues and predicted a poor prognosis

**FIGURE 4:**
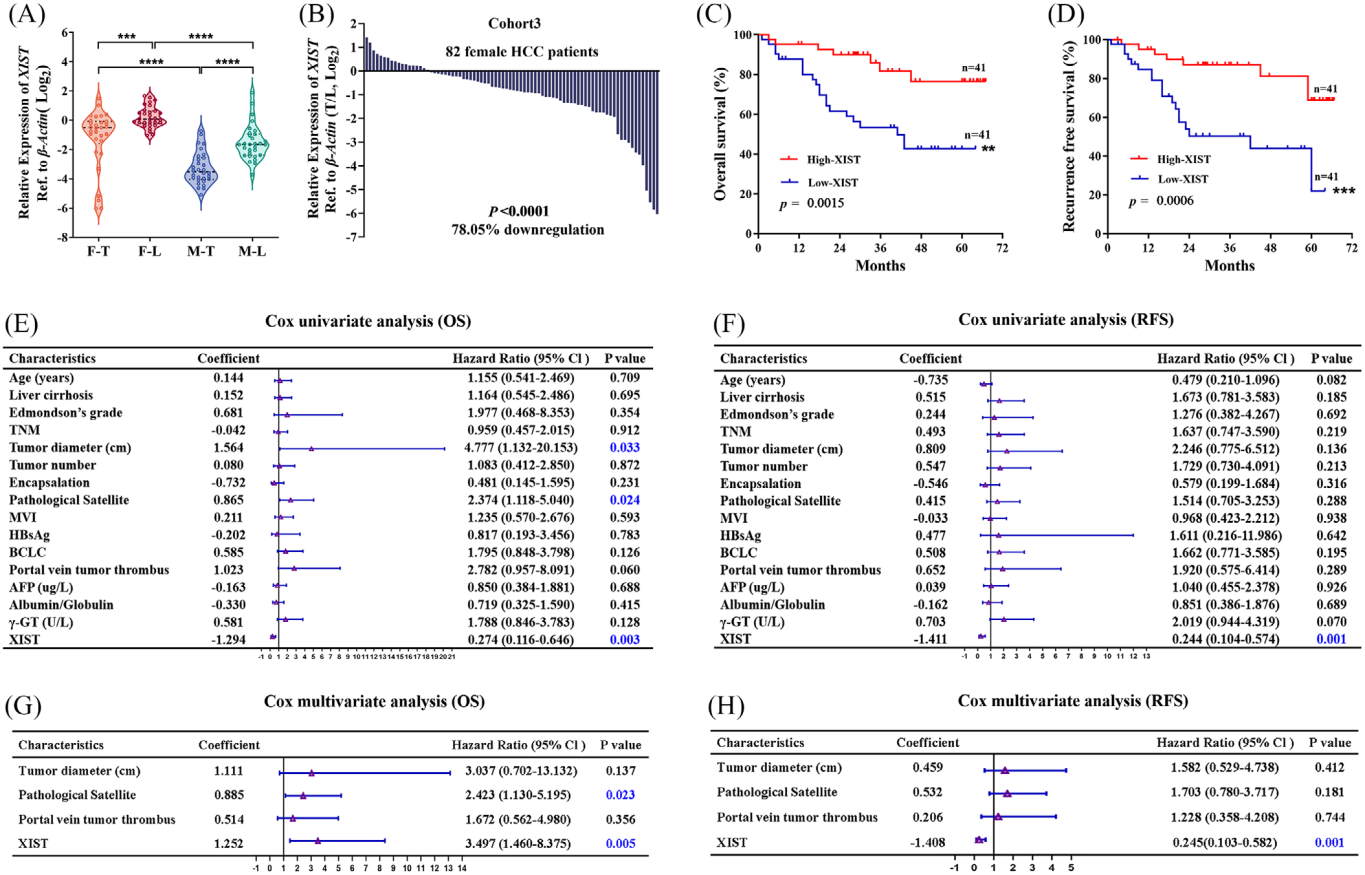
Differential expression of *XIST* in hepatocellular carcinoma (HCC) patients. (A) Expression of *XIST* in tumour tissues (T) and adjacent normal liver tissues (L) between genders (cohorts 1 and 2). (B) Expression of *XIST* in T and L (cohort 3). (C and D) Kaplan–Meier survival curves depicting overall survival (OS) (C) and recurrence‐free survival (RFS) (D) of HCC female patients with diverse *XIST* expression. (E) Cox univariate analysis of the factors related to OS of 82 female patients. (F) Cox univariate analysis of the factors related to RFS of 82 female patients. (G) Cox multivariate analysis of the factors associated with OS of 82 female patients. (H) Cox multivariate analysis of the factors associated with RFS of 82 female patients.

Subsequently, *XIST* expression level was firstly detected in 30 female HCC patients (cohort 1) and 30 male HCC patients (cohort 2). The clinical information samples of cohorts 1 and 2 are obtained in Tables S1 and S2. *XIST* was significantly downregulated in T compared with L in both HCC female and male patients (Figure 4A). Moreover, *XIST* was higher in females than males, whether in T or in L (Figure 4A). Considering the effect of *XIST* on XCI and the high expression abundance in female liver tissue, we further detected *XIST* in 82 HCC female patients (cohort 3, including cohort 1). *XIST* was significantly downregulated in F‐T compared with F‐L (Figure 4B).

Then, we tried to determine whether *XIST* in female HCC was associated with specific clinicopathological characteristics. Based on the median *XIST* expression in HCC tissues, the 82 female HCC patients were assigned to the high‐*XIST* expression group (*n* = 41) and low‐*XIST* expression group (*n* = 41). Although *XIST* did not have significant correlations with age, liver cirrhosis, Edmondson's grade, tumour‐node metastasis stage, tumour number, pathological satellite, microvascular invasion (MVI), HBsAg, plasma alpha‐fetoprotein level, albumin/globulin and γ‐glutamyl transpeptidase, but lower levels of *XIST* were associated with larger tumour diameters and poorer encapsulation status, and were also associated with more advanced Barcelona Clinic liver cancer stage and more portal vein tumour thrombosis (Table 1).

**TABLE 1:** Clinical characteristics of 82 hepatocellular carcinoma patients according to XIST expression levels.

	Lnc‐XIST			
Feature	High	Low	*χ* ^2^	*p*‐Value
All cases	41	41		
Age (years)			.0499	.8233
≤50	18	17		
>50	23	24		
Liver cirrhosis			.4802	.4884
Without	28	25		
With	13	16		
Edmondson's grade			2.216	.1366
I/II	6	2		
III/IV	35	39		
TNM			.0489	.825
I	20	19		
II/III	21	22		
Tumour number			.7343	.3915
1	35	32		
>1	6	9		
Tumour diameter (cm)			4.556	**.0328** ^*^
≤3	13	5		
>3	28	36		
Encapsulation			8.613	**.0033** ^**^
None or incomplete	29	39		
Complete	12	2		
Pathological satellite			.4564	.4993
Absent	23	26		
Present	18	15		
MVI			.497	.4808
Without	29	26		
With	12	15		
HBsAg			.213	.6444
Negative	3	2		
Positive	38	39		
BCLC			4.473	**.0344** ^*^
A	32	23		
B/C	9	18		
Portal vein tumour thrombus			3.905	**.0481** ^*^
Without	40	35		
With	1	6		
AFP (ug/L)			.0604	.8058
≤20	11	12		
>20	30	29		
Albumin/globulin			1.268	.2602
≤1.5	27	22		
>1.5	14	19		
γ‐GT (U/L)			.2253	.635
≤50	29	27		
>50	12	14		

*Note*: The median expression level was used as the cutoff. For analysis of correlation between XIST levels and clinical features, Pearson's chi‐squared tests were used. Results were considered statistically significant at *p* < .05.

Abbreviations: AFP, alpha‐fetoprotein; BCLC, Barcelona Clinic liver cancer staging system; Lnc‐XIST, long non‐coding RNA XIST; MVI, microvascular invasion; TNM, tumour‐node metastasis; γ‐GT, γ‐glutamyl transpeptidase.

Bold values represent significant differences.

* *p* < .05.

** *p* < .01.

Furthermore, as a result of Kaplan–Meier survival analysis, patients with higher *XIST* in HCC had a conspicuously better prognosis than those with lower *XIST*, with a higher OS (*p* = .0015) (Figure 4C) and more extended recurrence‐free survival (RFS) (*p* = .0006) (Figure 4D). A consistent conclusion was reached with our clinical results that female patients with higher *XIST* had better OS and RFS by using the Kaplan–Meier plotter database (https://kmplot.com/analysis/index.php?p = service&cancer = liver_rnaseq) (Figure S2).

Cox univariate analysis showed that tumour diameter, pathological satellite and *XIST* expression levels in HCC patients were conspicuously associated with OS (Figure 4E). Only the *XIST* expression level was conspicuously associated with RFS in HCC patients (Figure 4F). Cox multivariate analysis showed that *XIST* expression levels and pathological satellites were risk factors for OS and independent (Figure 4G). *XIST* expression levels also play the same role for HCC patients' RFS (Figure 4H).

Subsequently, we established stable *XIST*‐silenced HCC‐1016 cells by infecting *XIST*‐silenced lentivirus. *XIST* was validated by qPCR (Figure S3A). Results showed that *XIST* knockdown considerably promoted cell proliferation by Cell Counting Kit‐8 (CCK8) assay and EdU (5‐ethynyl‐2'‐deoxyuridine) immunofluorescence staining (Figure S3B–D). Previous study also found that targeted deletion of *Xist* in the blood compartment of mice can induce an aggressive, lethal blood cancer.^31^ Therefore, we believe that *XIST* may play a cancer‐suppressing role in HCC and contribute partially to the sex difference of HCC due to different liver expressions in different sexes.

### 3.4 *TET2* is significantly downregulated in HCC tissues

**FIGURE 5:**
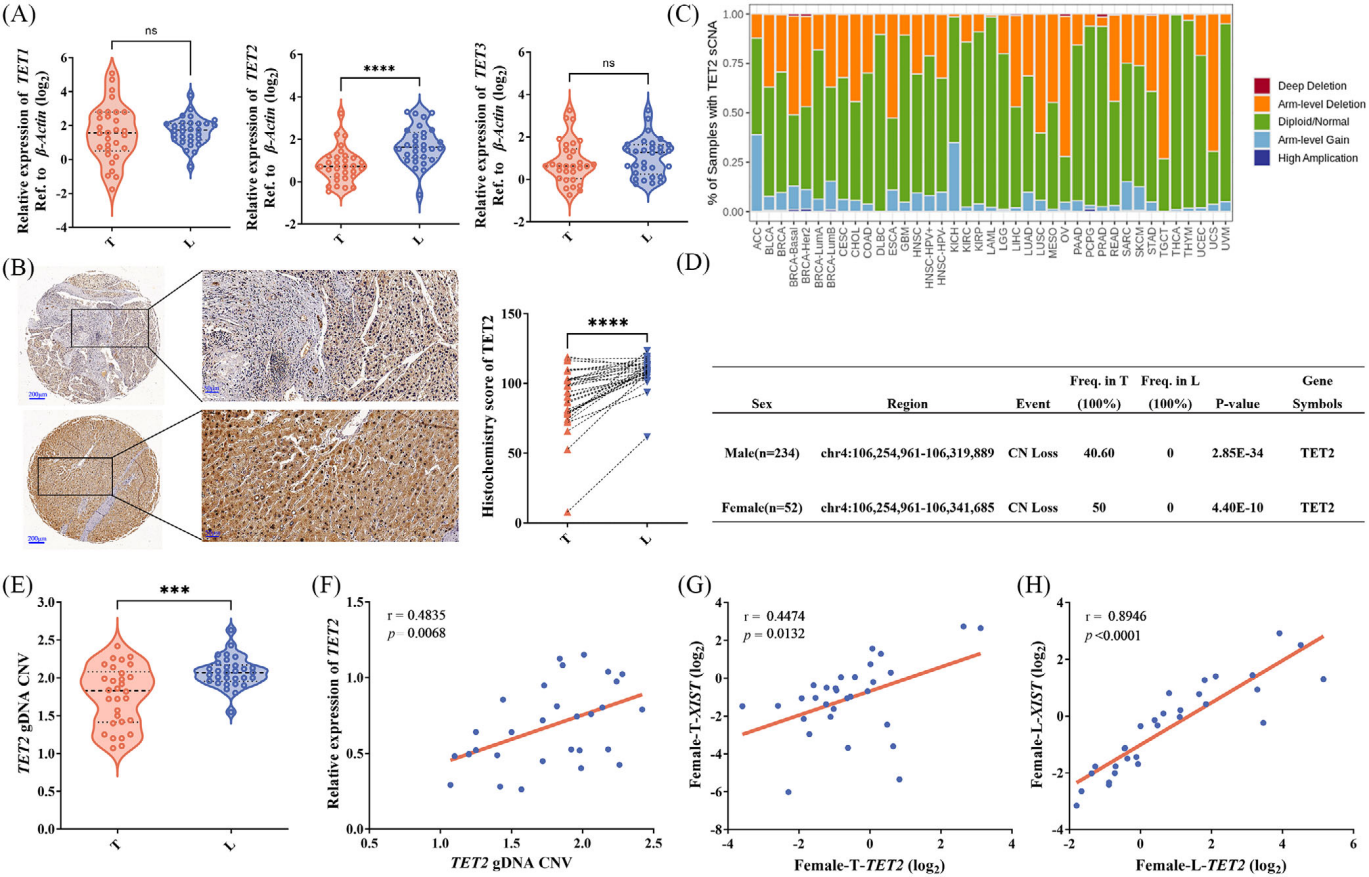
Tet methylcytosine dioxygenase 2 (TET2) is significantly downregulated in hepatocellular carcinoma (HCC) tissues. (A) Expression of *TET1*, *TET2* and *TET3* of tumour tissues (T) and adjacent normal liver tissues (L) in 30 females (cohort 1). (B) Histochemistry score of TET2 in 30 pairs females T and L (right) and representative samples (left). (C) The deletion of *TET2* sCNA in various cancer types was obtained from the TIMER2.0 database. (D) The data were analysed from GSE38323. (E) The copy number of *TET2* in cohort 1 tumour tissues and adjacent normal liver tissues. (F) Correlation between the *TET2* expression level and copy number in cohort 1 tumour tissues. Correlation between the expression level of *TET2* and *XIST* in cohort 1 tumour tissues (G) and adjacent normal liver tissues. (H) Pearson correlation analysis was used to measure the correlation.

Methyltransferases and demethylases (TETs) maintain the dynamic balance of DNA. TETs catalyse the oxidation of 5mC to 5hmC, which begins the demethylation of active DNA. Considering the decreased levels of 5hmC modification and the higher methylation levels in the first exon of *XIST* in female HCC tissues (Figures 1I,J and 2C–E), the expression of *TET1*, *TET2* and *TET3* were further examined in cohort 1. Only *TET2* was significantly downregulated in T compared with L (Figure 5A). The immunohistochemistry results also confirmed that TET2 was downregulated in HCC tissues (Figure 5B).

In recent years, some articles have reported that copy number variation can activate oncogenes and inactivate tumour suppressor genes, which are considered a key factor in many types of cancer, including liver cancer.^32^, ^33^, ^34^, ^35^ We found that the copy number of *TET2* was deficient in liver hepatocellular carcinoma (Figure 5C) by the TIMER2.0 database (http://timer.comp‐genomics.org/timer/). To explore the alteration of *TET2* genomic copy number in HCC, we used published data (GSE38323) to perform a data mining process.^36^ The results showed that deletion of the *TET2* genome was present in 50% of female HCC tissues (*p* = 4.40E‐10, *n* = 52), and deletion of the *TET2* genome occurred in 40.60% of male HCC tissues (*p* = 2.85E‐34, *n* = 234) (Figure 5D). We then assayed the copy number change of *TET2* in cohort 1 HCC samples (females) using TaqMan copy number. These results showed that the copy number of *TET2* genome is lacking in HCC tissues (Figure 5E), and its copy number was positively correlated with *TET2* mRNA expression level in HCC tissues (*r* = .4835, *p* = .0068, Figure 5F). In addition, the mRNA expression level of *TET2* and *XIST* in T and L from cohort 1 was detected. The result demonstrated that *XIST* expression level was positively correlated with *TET2* mRNA expression, whether in T (*r* = .4474, *p* = .0132, Figure 5G) or in L (*r* = .8946, *p* < .0001, Figure 5H). Therefore, we speculated that the downregulated expression of *TET2* was caused by the deletion of copy number, which might increase the methylation level of *XIST* first exon and lead to the downregulated expression of *XIST* in female HCC.

### 3.5 TET2 form complexes with YY1 binding to the promoter region of *XIST* and regulating the methylation level of *XIST*

**FIGURE 6:**
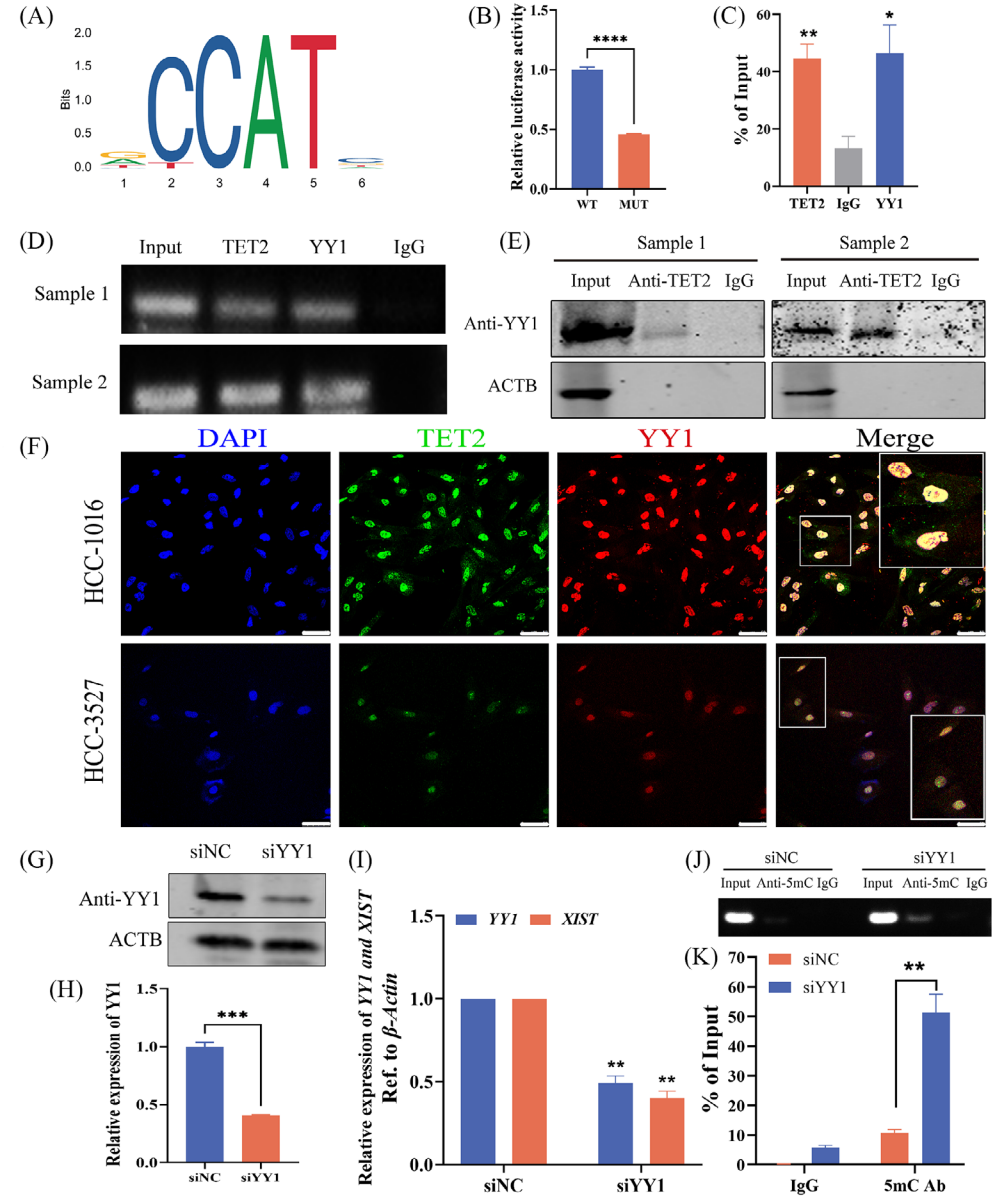
Tet methylcytosine dioxygenase 2 (TET2) forms complexes with YY1 binding to the promoter region of *XIST*. (A) Analysis the binding sites of YY1 in human *XIST* promoter region based on the JASPAR database (left) and the binding sites in the *XIST* promoter region (right, yellow indicates the detection sites, red font indicates the primers of *XIST* promoter region). (B) Fluorescence intensity expressed by dual luciferin of plasmid containing either the wild type (WT) or MUT. (C) Bar plot representing qPCR values of the chromatin immunoprecipitation (ChIP) results in female adjacent normal liver tissues. (D) *XIST* was quantified using DNA agarose gel electrophoresis. (E) Co‐immunoprecipitation (Co‐IP) of YY1 by TET2 was determined. The female adjacent normal liver tissues were immunoprecipitated with TET2 antibody, followed by western blotting assays for YY1 antibody. (F) Micrographs of representative immunofluorescence staining for TET2 (green) and YY1 expression (red) in hepatocellular carcinoma (HCC)‐1016 (up) and HCC‐3527 (down) cells. The arrow indicates colocalisation of TET2 and YY1. Scale bar, 50 μm. (G and H) The expression of YY1 in HCC‐1016 cells (knocked down YY1 in HCC‐1016 cells by transfecting siRNA) by western blot analysis. (I) Expression of YY1 and *XIST* in HCC‐1016 cells (knocked down YY1 in HCC‐1016 cells by transfecting siRNA) by qPCR. (J) *XIST* was quantified using DNA agarose gel electrophoresis. (K) The 5‐methylcytosine (5mC) level in the *XIST* promoter region by MeDIP assay and qPCR.

However, how does TET2 regulate the methylation level of *XIST*? A published study showed that loss of transcription factor YY1 could prevent *XIST* expression, and YY1 was bound solely to the unmethylated *XIST* allele.^37^ Consequently, we suspected whether TET2 could form complexes with YY1 binding to the promoter region of *XIST* and regulating the methylation level of *XIST*. Firstly, to validate whether YY1 directly binds to the *XIST* promoter, the JASPAR database was analysed for YY1 binding sites within the *XIST* gene promoter sequences (Figure 6A). Then, the dual‐luciferase reporter plasmid system was constructed which contained the full length of *XIST* promoter region containing YY1 binding sites (WT) or lacking YY1 binding sites (MUT). The results showed that the plasmids co‐transfected with YY1 and *XIST* promoter (MUT) could significantly decrease the expression abundance of luciferase in HCC‐1016 cell line (Figure 6B). It suggested that YY1 could bind to the promoter of *XIST* and promote transcription. Moreover, we performed a ChIP‐qPCR assay in female adjacent normal liver tissues. The results demonstrated that TET2 and YY1 could specifically bind to the *XIST* promoter (Figure 6C,D). Furthermore, physical contact between TET2 and YY1 was further suggested by Co‐IP experiments performed using female adjacent normal liver tissues. It was shown that these two proteins could be co‐precipitated (Figure 6E). In addition, the spatial distribution of TET2 and YY1 coincides and interacts with each other by immunofluorescence assays in HCC‐1016 and HCC‐3527 cells (Figure 6F). To demonstrate whether the TET2–YY1 complex can regulate the expression of *XIST*, we knocked down YY1 in HCC‐1016 cells by transfecting siRNA (Figure 6G,H). The result showed that knockdown YY1 significantly downregulated the expression level of *XIST* (Figure 6I), and the methylation level of its promoter region was increased by MeDIP analysis (Figure 6J,K). Taken together, TET2 could form complexes with YY1 binding to the promoter region of *XIST* in female cells and regulating the methylation level of *XIST*, thereby affecting the expression of *XIST*.

### 3.6 TET2 functions as a tumour suppressor in hepatocarcinogenesis

**FIGURE 7:**
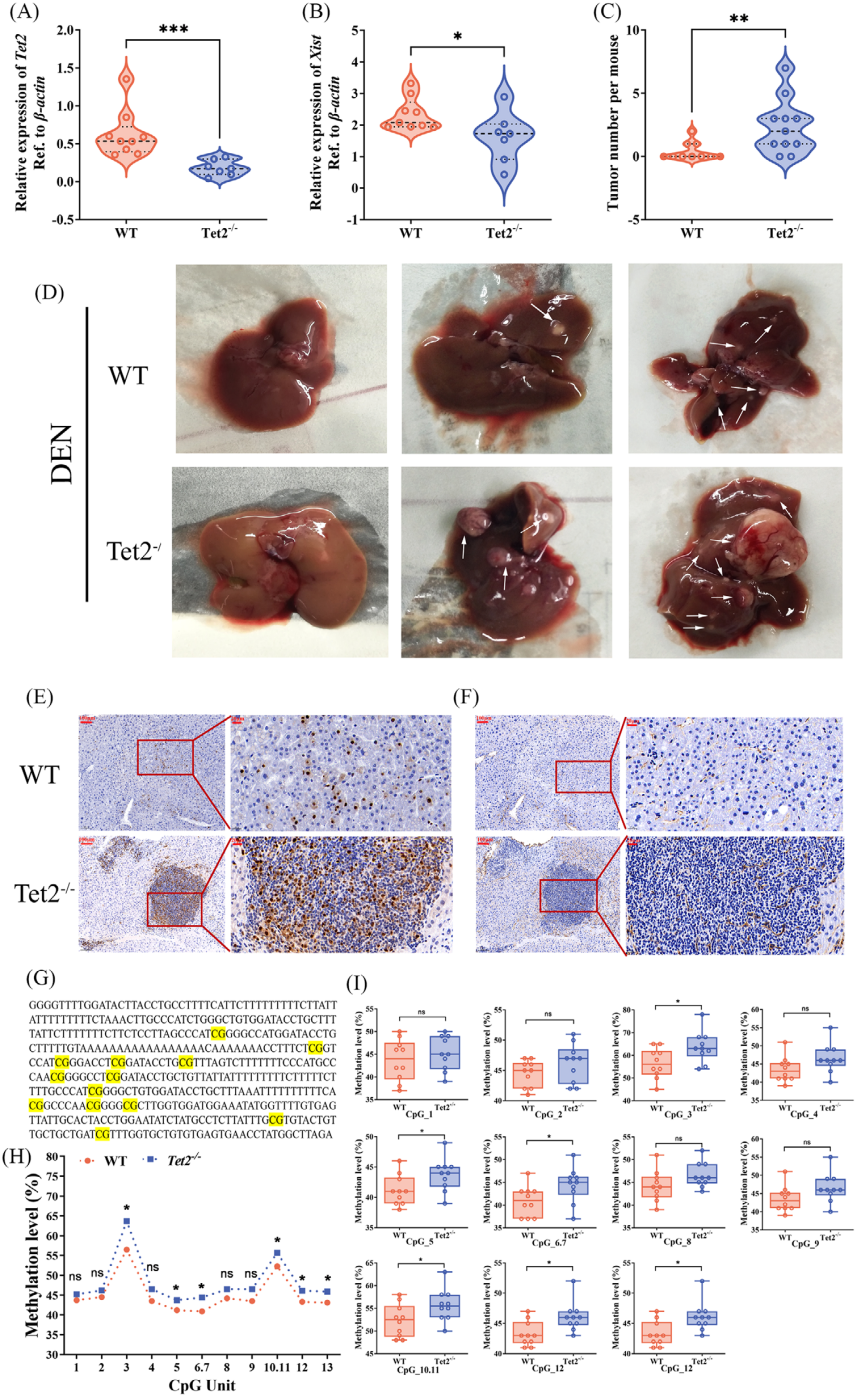
Tet methylcytosine dioxygenase 2 (TET2) plays a protective role in hepatocellular carcinoma (HCC). Expression of *Tet2* (A) and *Xist* (B) in female wild‐type (WT) mice (*n* = 9) and *Tet2* ^−/−^ mice (*n* = 7). (C) The tumour number per mice in diethylnitrosamine (DEN)‐administered female mice. (D) Representative pictures of the livers (arrows depict tumours). Immunohistochemistry (IHC) detection and quantification of Ki67 (E) and CD31 (F) protein expression in tumours from DEN‐administered female mice. (G) Methylation mass spectrometry detection product sequence of *Xist* first exon region (yellow indicates the detection sites, a total of 13). (H) Mean methylation levels of CpG sites in *Xist* first exon region in liver tissues between 10 WT mice and 10 *TET2* ^−/−^ mice of females. (I) Methylation level of each CpG site between WT mice and 10 *TET2* ^−/−^ mice of female in *Xist* first exon.

To explore the role of TET2 in hepatocarcinogenesis, *Tet2* ^−/−^ mice were constructed. The expression level of *Tet2* and *Xist* in the liver tissue of female mice was detected. Figure 7A,B indicates that *Tet2* and *Xist* in *Tet2* ^−/−^ mice were conspicuously lower than in WT mice. Moreover, we established a chronic DEN model by injecting liver carcinoma pro‐DEN into WT and *Tet2* ^−/−^ female and male mice and sacrificing them 40 weeks after DEN administration. The number of tumours in each female mouse was measured. Macroscopic liver analysis revealed that DEN‐administered *Tet2* ^−/−^ female mice had more tumours than in controls (Figure 7C,D), and tumour incidence in male DEN‐induced mice was remarkably higher than in female mice (Figure S4 and Table S5). Next, the protein expression of cell proliferation (Ki67) and vessel density (CD31) in the liver cancer tissues from DEN‐administered female *Tet2* ^−/−^ mice and WT mice were examined. It exhibited a significantly increased proliferation rate and tumour vessel density in the liver cancer tissues compared with the DEN‐administered WT mice (Figure 7E,F). Methylation of CpG sites of the *Xist* first exon region in liver tissues between 10 WT and 10 *Tet2* ^−/−^ female mice was quantified by Sequenom MassARRAY methylation profiling. All 13 CpG sites in the first exon region were divided into 11 CpG site units (Figure 7G). As shown in Figure 7H,I, methylation levels in *Tet2* ^−/−^ mice were conspicuously increased over the corresponding methylation levels in WT mice. All results suggested that TET2 in hepatocytes could inhibit DNA methylation modifications in the first exon region of *XIST* and maintain high levels of *XIST* expression in female liver tissues, thereby inhibiting tumourigenesis.

## 4 DISCUSSION

As our study confirmed, the 5hmC distribution was significantly different between female and male HCC patients. The number of 5hmC reads in the first exon of *XIST* from female tumour tissues were remarkably lower than in female adjacent liver tissues. On further analysis, *XIST* was downregulated in tumour tissues, which in females was higher than in males. *XIST* was positively correlated with OS and RFS in female HCC tissues. We found that TET2‐mediated DNA demethylation is vital in the molecular mechanism of *XIST* expression. TET2 achieves *XIST*’s DNA hypomethylation state by binding to the YY1 transcription factor in female normal liver tissue. Furthermore, the decrease of TET2 in female HCC tissue was owing to the loss of *TET2* copy number.

Epidemiological data suggest that males had a greater number of liver cancer than females. Generally, the androgen axis promotes hepatocarcinogenesis, while the estrogen axis acts as a tumour suppressor. However, clinical trials of sex hormone‐specific therapies for HCC have not yielded satisfactory results.^38^, ^39^ There are even some conflicting findings. Ligand‐activated androgen receptors may inhibit HCC metastasis by activating the p38 pathway to induce apoptosis.^40^ It has also been shown that androgen receptors could improve cell adhesion by activating the AKT signalling pathway, thereby reducing cell migration.^41^ It can be seen that there may be deeper reasons behind the gender differences in HCC.

In addition to sex hormones, there is a very different event between the two sexes—XCI. lncRNA *XIST* regulates the formation of XCI in female mammals to balance the X‐linked gene expression between sexes.^27^, ^42^ However, XCI is incomplete, with approximately 23% of genes escaping from XCI, causing higher expression levels in XX female XCI escapers than in XY males.^29^ Our results indicate that the sex dimorphism associated with *XIST* in hepatocarcinogenesis is mainly reflected in the presence of two X chromosomes in female hepatocytes, one of which achieves XCI under DNA methylation regulation.^43^ *XIST* is an XCI escape gene that needs to be highly expressed in hepatocytes to help achieve XCI in female cells, and its promoter region (including the first exon region) has a lower level of DNA methylation. The DNA methylation level of the *XIST* promoter region (including the first exon region) is significantly increased in female HCCs (Figure 2C–E), leading to a decrease in its expression level (Figure 4B). In male hepatocytes, there is only one X chromosome, which does not require XCI. The DNA methylation level of the *XIST* promoter region (including the first exon region) is already relatively high (Figure 3B–D) in male hepatocytes. No significant changes were found in the comparison between male hepatocytes and HCC cells (Figure 3B–D). These escapes from XCI may contribute to gender‐biased disease.^10^, ^44^ Perhaps, genes that escape from XCI not only contribute to achieving XCI,^45^ but also have other functions, such as inhibiting tumour development.^46^ Compared to males, female hepatocytes need to reduce the high expression of XCI escape genes (such as *XIST*), which is actually equivalent to a higher HCC threshold for females than males. Our results suggest that the low expression of TET2 caused by genome copy number deletion in female HCC cells may downregulate the expression of *XIST* through DNA demethylation.

Why this regulatory relationship does not hold true in male hepatocytes? Our identified transcription factor YY1, which interacts with TET2, may play a crucial role in this process. Published study has shown that YY1 only binds to unmethylated *XIST* alleles.^37^ Our results indicate that high methylation levels in the *XIST* promoter region (Figure 3B–D) may hinder the binding of YY1, and the binding of TET2 in this region naturally decreases in male hepatocytes (Figure S5), and the expression level of TET2 naturally does not regulate the expression of *XIST*. The decrease in *XIST* expression in male HCC tissues (Figure 4A) may be caused by other reasons, such as RNA post‐transcriptional modifications, etc.^47^, ^48^ In female hepatocytes, the *XIST* promoter region is in a low methylation state (Figure 2C–E), and YY1–TET2 complex may bind in this region, maintaining the low methylation state. In female HCC cells, low expression of TET2 is caused by copy number deletion (Figure 5), resulting in a decrease in 5hmC levels and an increase in 5mC levels in this region, thereby reducing the expression of *XIST*. It can be seen that the 5hmC and 5mC states of the XCI escape gene *XIST* promoter region are inconsistent, which may be the reason for the different susceptibility of males and females to HCC.

The role of *XIST* on tumourigenesis and progression is complicated and indistinguishable. Yildirim et al. have demonstrated that deleting *XIST* is sufficient to induce an aggressive, lethal blood cancer in mice.^31^ A recent study showed that loss of *XIST* hampers mammary stem cell differentiation and promotes tumourigenesis.^46^ Some researchers have explicitly proposed that *XIST* is involved in carcinogenesis and tumour suppressor pathways in tumour pathology.^49^ There were also many studies indicating that *XIST* had the role of the oncogene. An analysis of published sequencing data on cancer tissues revealed that *XIST* was activated and expressed in various male tumours characteristics of XCI.^30^ Much evidence shows that *XIST* plays a vital role in the proliferation, invasion, migration, apoptosis and chemosensitivity of non‐small‐cell lung cancer (NSCLC) cells.^50^, ^51^ *XIST*’s role in HCC is even more chaotic. *XIST* was less expressed in HCC tissues and inhibited HCC cell proliferation and metastasis by specifically regulating miR‐92b.^12^, ^52^, ^53^ It has also reported that *XIST* was upregulated in HCC tissues.^54^, ^55^, ^56^ The reason may be that some studies did not accurately distinguish the gender of tissue sample sources (some studies put together the data of male and female patients), or the sample size was too small. In our research, detection data from male and female HCC tissues were processed and analysed separately (Figure 4A). Results confirmed that the expression of *XIST* in females was much higher than in males. In a large sample size (*n* = 82) cohort of female patients with HCC, we further clarified that *XIST* was downregulated in HCC tissues (Figure 4B). Female patients with lower *XIST* expression had significantly poorer prognoses than those patients with higher *XIST* expression (Figure 4C–H). Downregulating *XIST* expression considerably promoted cell proliferation (Figure S3B–D). From the above results, it can be speculated that *XIST* may work like a tumour suppressor in female HCC.

How to regulate the transcriptional activation of *XIST* is also an interesting issue for researchers. With the deepening of XCI research, lncRNA regulators targeting *XIST* have been discovered, such as *JPX* and *TSIX*, which can promote or inhibit, expanding the scope of XCI research.^57^, ^58^ However, people do not know much about the regulatory mechanism of *XIST* expression under pathological conditions. Thanks to the SMART database, we established the relationship between the level of DNA methylation and *XIST* in female HCC tissues but not in males.

In summary, this study shows that the downregulation of *XIST* expression can lead to poor prognosis in female HCC, and TET2 can form a complex with YY1 and bind to the *XIST* promoter region, maintain the hypomethylation of this region, as well as promote *XIST* expression. All the above results suggest that *XIST* may act as tumour suppressor in female hepatocarcinogenesis. Of course, our study also has study limitations, such as the clinical samples only derived from the same hospital. Moreover, it is unknown whether the decreased expression of *XIST* induces HCC in vivo. We cannot definitively state that *XIST* down‐expression in female HCC cases is causal for HCC. The questions will be explored in the following study.

## 5 CONCLUSIONS

This study found that the expression level of *XIST* regulated by the YY1–TET2 complex is associated with the prognosis of female HCC patients. These results showed that *XIST* may act as a tumour suppressor gene in female hepatocarcinogenesis. A better understanding of the XCI regulatory network may help to develop relevant targeted therapy for clinical treatment HCC in females.

## AUTHOR CONTRIBUTIONS

Fu Yang, Hui Liu and Shuhan Sun designed the study. Zhihui Dai and Sijie Wang performed data analysis and prepared the manuscript. Xinggang Guo collected and analysed clinical data. Yuefang Wang, Haozan Yin, Chenyang Mu and Jan Tan performed the experiments. All authors approved the submitted version.

## CONFLICT OF INTEREST STATEMENT

The authors declare they have no conflicts of interest.

## ETHICS STATEMENT

This research was approved by the ethics committee of the Naval Medical University. All patients were consented by an informed consent process.

## Supporting information


**Figure S1** The correlation between the methylation level and the relative expression of *XIST* in hepatocellular carcinoma (HCC) tissues using the Shiny Methylation Analysis Resource Tool (SMART) date. The correlation between the methylation level and the relative expression of *XIST* in 118 female (A) and in 244 male (C) HCC tissues (probe:cg12653510). The correlation between the methylation level and the relative expression of *XIST* in 113 female (B) and in 235 male (D) HCC tissues (probe:cg03554089). The correlation of female date was measured by Pearson correlation analysis. The correlation of male date was measured by Spearman correlation analysis.

**Figure S2** The prognosis of patients with higher expression of *XIST* are better from the database of Kaplan–Meier plotter. (A) The overall survival of 118 female liver cancer patients. (B) The recurrence‐free survival of 106 female liver cancer patients. (C) The overall survival of 246 male liver cancer patients. (D) The recurrence‐free survival of 210 male liver cancer patients.

**Figure S3** XIST suppresses hepatocellular carcinoma (HCC)‐1016 cell proliferation. (A) Relative expression level of XIST in XIST‐silenced HCC‐1016 cells compared with control cells determined by real‐time polymerase chain reaction (PCR). (B) Proliferation of HCC‐1016 cells assessed by CCK8 assay. XIST silencing promote cell proliferation. (C) EdU immunofluorescence staining of HCC‐1016 cells. (D) The percentage of EdU‐positive nuclei.

**Figure S4** Representative pictures of the livers (arrows depict tumours) from diethylnitrosamine (DEN)‐administered male mice.

**Figure S5** TET2 and YY1 could not binding to the promoter region of *XIST* in male adjacent normal liver tissues by chromatin immunoprecipitation (ChIP)‐qPCR. (A) Bar plot representing qPCR values of the ChIP results in male adjacent normal liver tissues. (B) *XIST* was quantified using DNA agarose gel electrophoresis.

Supporting Information

Supporting Information

Supporting Information

Supporting Information

Supporting Information

Supporting Information

## Data Availability

Data supporting this study will be made available upon reasonable request.
