# Supplementary figures and images for: Gender dimorphism in hepatocarcinogenesis—DNA methylation modification regulated X‐chromosome inactivation escape molecule XIST
Source: Clin Transl Med. 2023 Dec 26;13(12):e1518. doi: 10.1002/ctm2.1518 (PMC10751514; doi:10.1002/ctm2.1518)

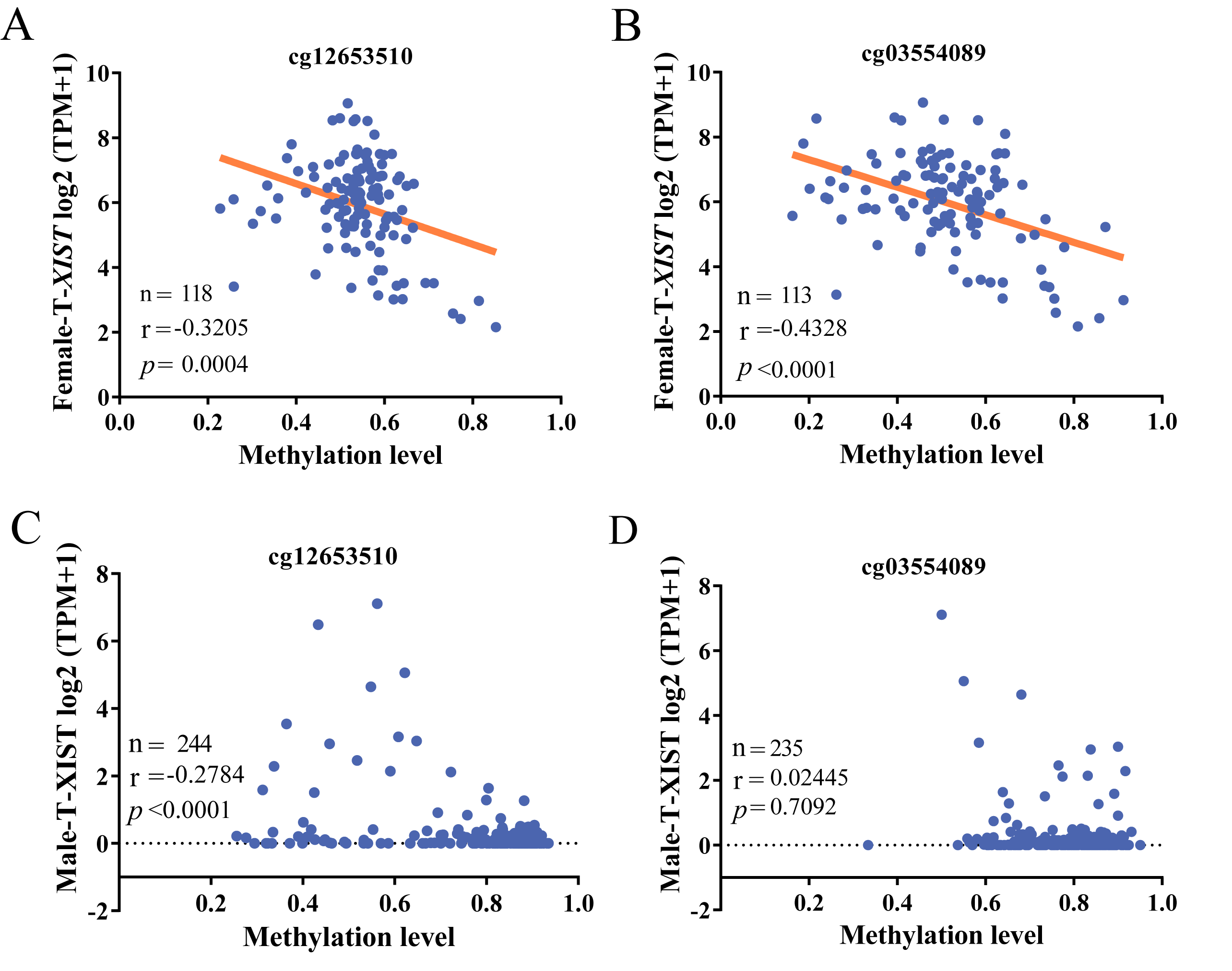

Supplement: Supplementary file 1 — Figure S1 The correlation between the methylation level and the relative expression of XIST in hepatocellular carcinoma (HCC) tissues using the Shiny Methylation Analysis Resource Tool (SMART) date. The correlation between the methylation level and the relative expression of XIST in 118 female (A) and in 244 male (C) HCC tissues (probe:cg12653510). The correlation between the methylation level and the relative expression of XIST in 113 female (B) and in 235 male (D) HCC tissues (probe:cg03554089). The correlation of female date was measured by Pearson correlation analysis. The correlation of male date was measured by Spearman correlation analysis. [file CTM2-13-e1518-s004.tif]

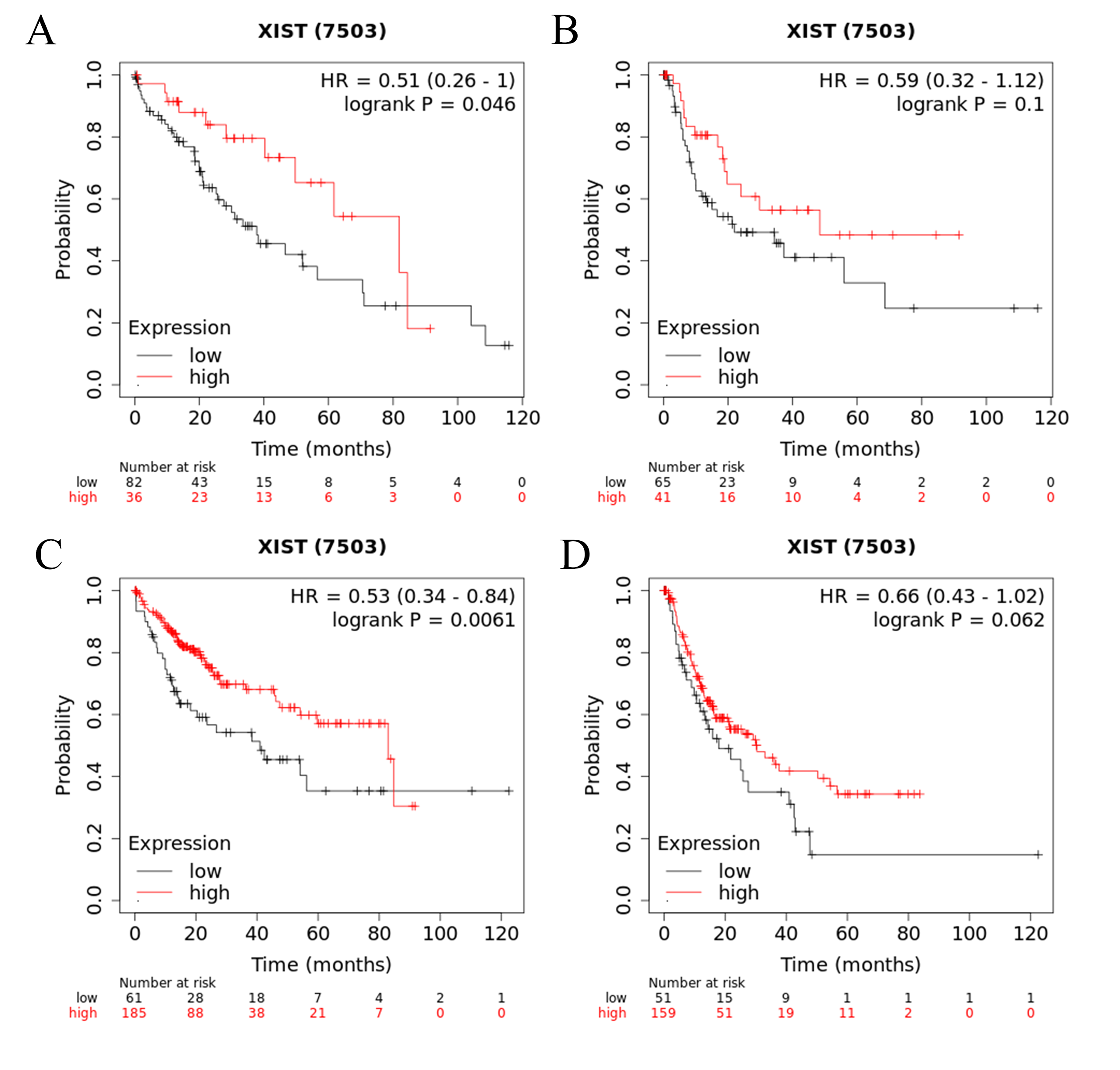

Supplement: Supplementary file 2 — Figure S2 The prognosis of patients with higher expression of XIST are better from the database of Kaplan–Meier plotter. (A) The overall survival of 118 female liver cancer patients. (B) The recurrence‐free survival of 106 female liver cancer patients. (C) The overall survival of 246 male liver cancer patients. (D) The recurrence‐free survival of 210 male liver cancer patients. [file CTM2-13-e1518-s005.tif]

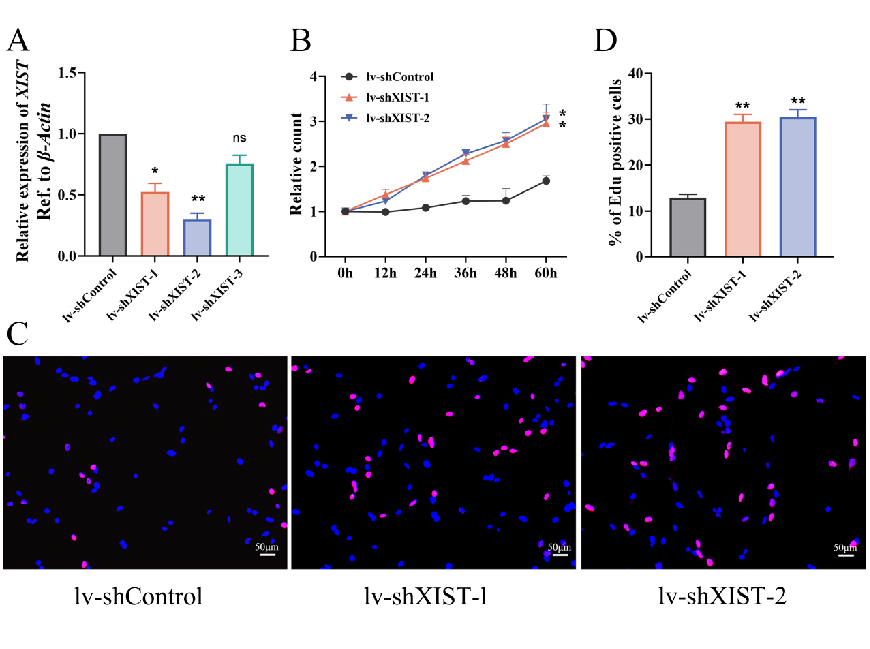

Supplement: Supplementary file 3 — Figure S3 XIST suppresses hepatocellular carcinoma (HCC)‐1016 cell proliferation. (A) Relative expression level of XIST in XIST‐silenced HCC‐1016 cells compared with control cells determined by real‐time polymerase chain reaction (PCR). (B) Proliferation of HCC‐1016 cells assessed by CCK8 assay. XIST silencing promote cell proliferation. (C) EdU immunofluorescence staining of HCC‐1016 cells. (D) The percentage of EdU‐positive nuclei. [file CTM2-13-e1518-s008.tif]

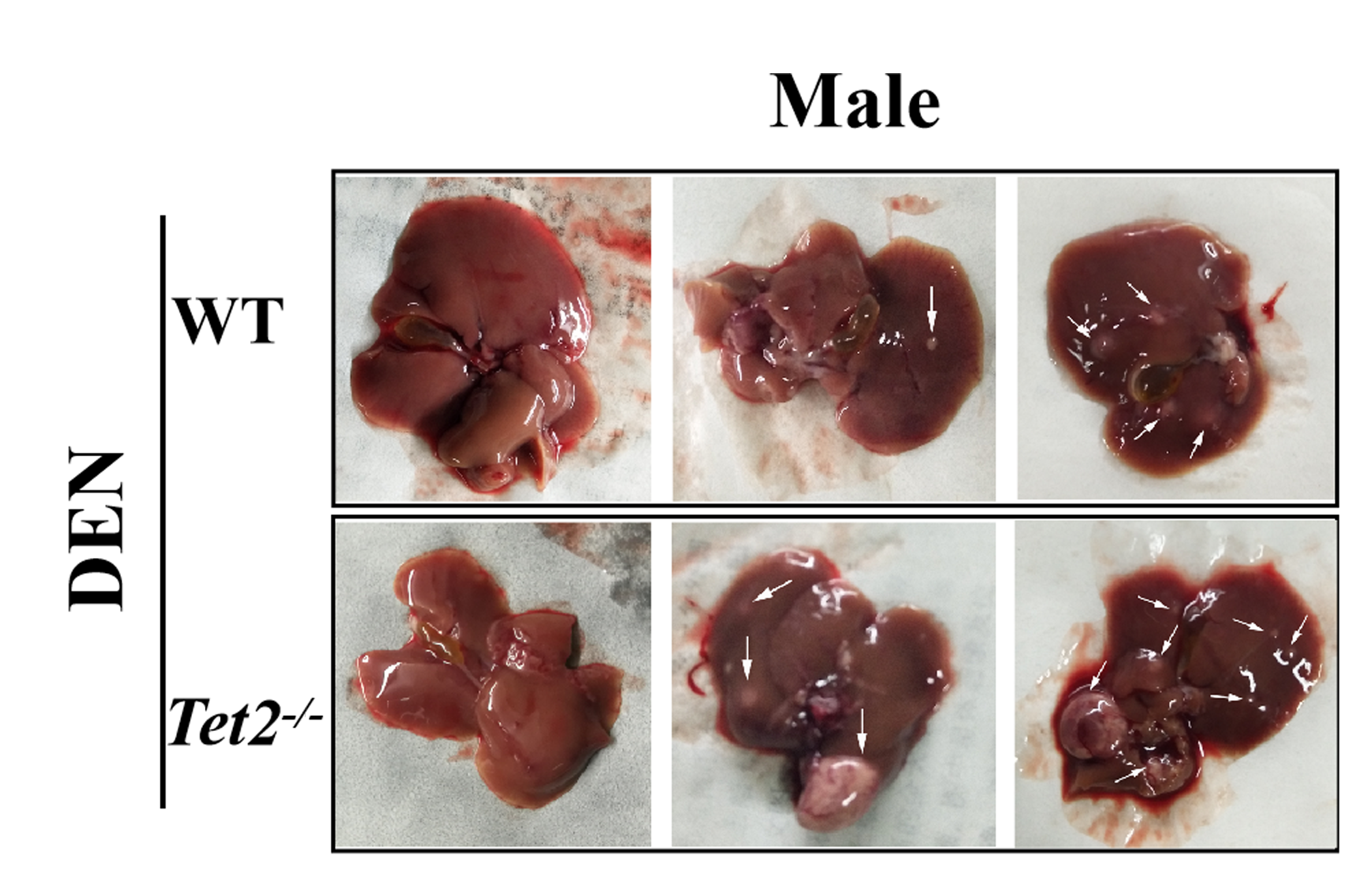

Supplement: Supplementary file 4 — Figure S4 Representative pictures of the livers (arrows depict tumours) from diethylnitrosamine (DEN)‐administered male mice. [file CTM2-13-e1518-s009.tif]

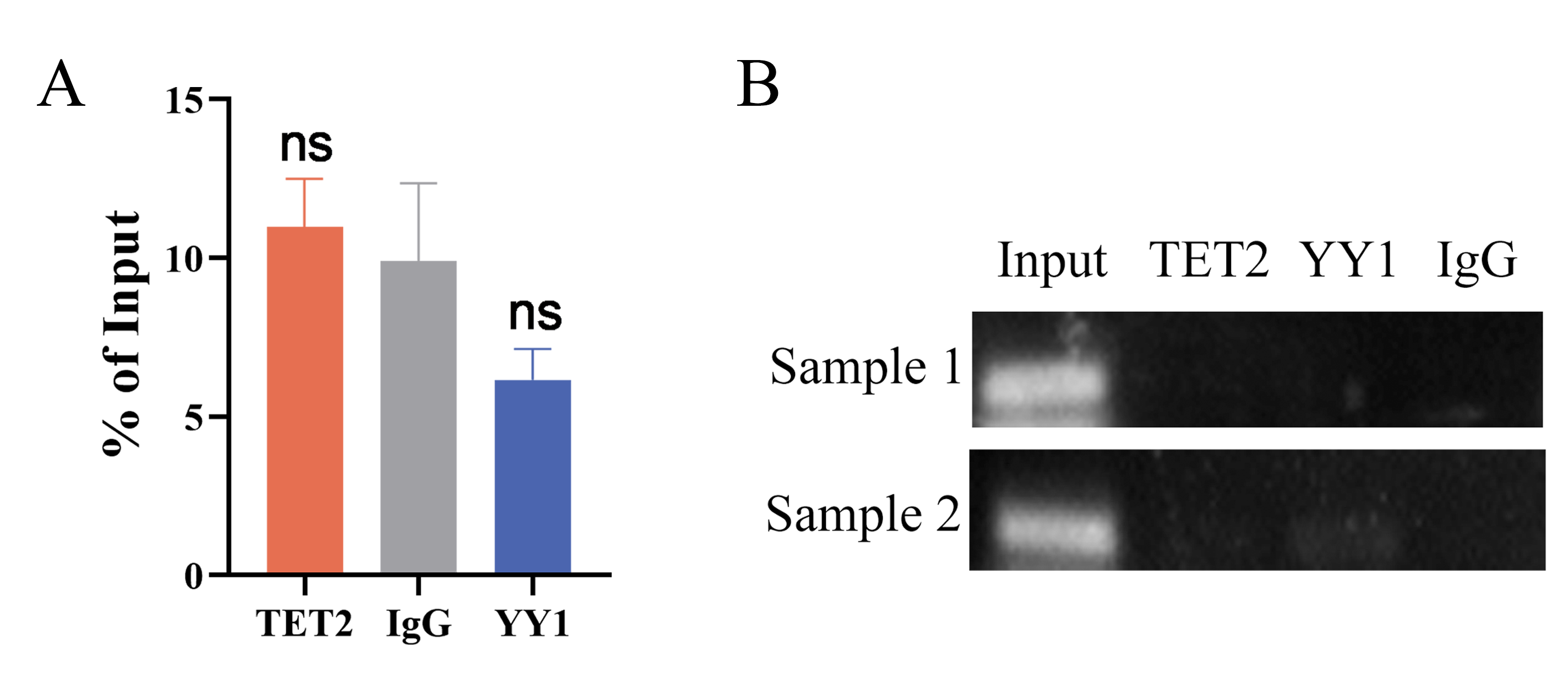

Supplement: Supplementary file 5 — Figure S5 TET2 and YY1 could not binding to the promoter region of XIST in male adjacent normal liver tissues by chromatin immunoprecipitation (ChIP)‐qPCR. (A) Bar plot representing qPCR values of the ChIP results in male adjacent normal liver tissues. (B) XIST was quantified using DNA agarose gel electrophoresis. [file CTM2-13-e1518-s007.tif]
