# Supplementary material for: Gender dimorphism in hepatocarcinogenesis—DNA methylation modification regulated X‐chromosome inactivation escape molecule XIST
Source: Clin Transl Med. 2023 Dec 26;13(12):e1518. doi: 10.1002/ctm2.1518 (PMC10751514; doi:10.1002/ctm2.1518)
Supplement: Supplementary file 11 — Supporting Information [file CTM2-13-e1518-s003.docx]

1. XIST promoter(WT) sequence 2200bp

>hg38_refGene_NR_001564 range=chrX:73820651-73854753 5'pad=0 3'pad=0 strand=-repeatMasking=none

taccatttaaaaaactccaatgacaaccaatgagagtgatctgctagcgtggctgcccacctggaaccagggcctggtcacagaaacccaggacaaccagtttataaattgtctttttgcctttttatttgaaaaggtatttttgtgaaacaaaaggttttaat

ttcagagtcaaatttatcaatcttttatatttgatgctacttgagtctttagaaaatttttccccaactaaatttctttttt

ctttttttgagatggagtcttgctctgtcgcccgggctggagtgcagtggcaccatctcggttcactgcaacctccgcctcc

tggattcaagtgattctcctgcctcagcctcccaagtagctgggattataggtgcctgccaccatgcccggctaatttttgt

atttatagtagagacggggtttcgctatgttggccaggctggtctcaaactcctcacctcaggtgatccagccacctcggcctcccaaagtgctgggattacaagcatgagccaccgcgcccggccaaccaaatttcttaaaagtatagaacatttccacacttgtagaaacttctagtagataaagttgtattagaggtttggtaatccagtctttctgatagctgcttcataggtgtcactgtaggtagtttctctctttgttaagcattgttttctcaaaggtaccattgtattgcaccactttacaatacaataaccctcattt

ggaatgacaagtcagattactttttttgtttgatccaaggacacttagaagatggaaggtatttcttaaggaagaaaatttgcatactttattatttttctggaaactgaagagcaagcttacctgatagatttgggtcgatagttacactttccagttcagcc

agtgggagggtaatgtatcctttgactttctagaagttgctctataatacttaagttgccattgtctcccttctggttctct

gttgtttctcgaatgacatagaagagtttatgagattaattgcttcctactgaaatagccaggtttctgagggcagctttgg

cttttatatgagctttttcttcctaccacttatcagttactgacagagggtcaacttcactactgtgtcatatggttggtcc

aatgttgggttcctcagattcatggaggaaataggaaaatagtaaagattaaggagtcatagttaaaaaattacaaacaggtcacaaacagtactctttcttgattatttaggaaccaaatagccattctatgaaatgtctttctttcctttttctctcttgct

caccaattgactcgtaagcacttccgttctcttatggttgggagccatacaaggtagagtgttggagtaaaaactacattgactctgaatcatggtgtgtgacctcgggcacatgatgtaacctctataggactctattttaatgtataaaacaggaataatcctttattattatctatgcaatacatattccattatctattacatgggataatggaatgggaagtcccttgaagatggtactaa

cctcaatgtattactcctttctagcttctttggttcaaaagtttggtggaggagttacaaattctggtttgaatgatatatt

tggatactttatcaacacatcaaagctctacctatcccttcccccattctcaaaaccaagctgaattaacatctttacattt

attatgcagtttatggaggattttagcattaattattgcttgatttactcaatgtccccatgttatagatgagaactggaaa

acccattgaagttgtgactcctggtctagaaatgaagtctacttccagttaatgttctttctggtatgtctttgctttcttg

aaatttcccttttttgtccttactgggtaaattttgaaccaaccaaatcacaaagatgtccggctttcaatcttctaggcca

cgcctcttatgctctctccgccctcagcccccCCTTCAGTTCTTAAAGCGCTGCAATTCGCTGCTGCAGCCATATTTCTTACTCTCTCGGGGCTGGAAGCTTCCTGACTGAAGATCTCTCTGCACTTGGGGTTCTTTCTAGAACATTTTCTAGTCCCCCAACACCCTTTATGGCGTATTTCTTTAAAAAAATCACCTAAATTCCATAAAATATTTTTTTAAATTCTATACTT

1. XIST promoter(MUT) sequence 1991bp

>hg38_refGene_NR_001564 range=chrX:73820651-73854753 5'pad=0 3'pad=0 strand=-repeatMasking=none

ttaaaaaactccaatgacaaccaatgagagtgatctgctagcgtggctgcccacctggaaccagggcctggtcacagaaacccaggacaaccagtttataaattgtctttttgcctttttatttgaaaaggtatttttgtgaaacaaaaggttttaatttcaga

gtcaaatttatcaatcttttatatttgatgctacttgagtctttagaaaatttttccccaactaaatttcttttttcttttttttgctctgtcgcccgggctggagtgcagtgttcactgcaacctccgcctcctggattcaagtgattctcctgcctcagcctcccaagtagctgggattataggtgcctgcccccggctaatttttgtatttatagtagagacggggtgccaggctggtctcaaactcctcacctcaggtgatccagccacctcggcctcccaaagtgctgggattacaagcatgagccaccgcgcccggccaacc

aaatttcttaaaagtatagaacatttccacacttgtagaaacttctagtagataaagttgtattagaggtttggtaatccag

tctttctgatagctgcttcataggtgtcactgtaggtagtttctctctttgttaagcattgttttctcaaaggtgtattgca

ccactttacaatacaataaccctcatttggaatgacaagtcagattactttttttgtttgatccaaggacacttatatttct

taaggaagaaaatttgcatactttattatttttctggaaactgaagagcaagcttacctgatagatttgggtcgatagttac

actttccagttcagccagtgggagggtaatgtatcctttgactttctagaagttgctctataatacttaatcccttctggtt

ctctgttgtttctcgaatgacatagaagagtttatgagattaattgcttcctactgaaatagccaggtttctgagggcagct

ttggcttttatatgagctttttcttcctaccacttatcagttactgacagagggtcaacttcactactgtgtcatggtccaa

tgttgggttcctcagattggaaataggaaaatagtaaagattaaggagtcatagttaaaaaattacaaacaggtcacaaacagtactctttcttgattatttaggaaccaatgaaatgtctttctttcctttttctctcttgctcaccaattgactcgtaagca

cttccgttctcttgggacaaggtagagtgttggagtaaaaactacattgactctgaatgtgtgacctcgggcacatgatgtaacctctataggactctattttaatgtataaaacaggaataatcctttattattatctatgcaatacatatatctattaataa

gtcccttaacctcaatgtattactcctttctagcttctttggttcaaaagtttggtggaggagttacaaattctggtttgaa

tgatatatttggatactttatcaacacatcaaagctctacctatcccttccctcaaaaccaagctgaattaacatctttaca

tttattatgcagttggattttagcattaattattgcttgatttactcaattagatgagaactggaaaagaagttgtgactcc

tggtctagaaatgaagtctacttccagttaatgttctttctggtatgtctttgctttcttgaaatttcccttttttgtcctt

actgggtaaattttgaaccaaccaaatcacaaagatgtccggctttcaatcttctaggccacgcctcttatgctctctccgc

cctcagcccccCCTTCAGTTCTTAAAGCGCTGCAATTCGCTGCTCTTACTCTCTCGGGGCTGGAAGCTTCCTGACTGAAGATCTCTCTGCACTTGGGGTTCTTTCTAGAACATTTTCTAGTCCCCCAACACCCTTGTATTTCTTTAAAAAAATCACCTAAATAAATATTTTTTTAAATTCTATACTT

**Supplementary Materials and methods**

**Total RNA extraction, cDNA synthesis and quantitative real-time PCR**

Total RNA was extracted from frozen tissue using TRIzol reagent (Takara, Dalian, China). The concentration and purity of total RNA were measured by Nanodrop 2000 spectrophotometer (ThermoFisher Scientific, Inc.). We only further analyzed samples with value of OD260/OD280 between 1.8 and 2.0. cDNA synthesis was performed following the experimental procedure of the RR036A reagent kit (Takara, Dalian, China). Quantitative real-time PCR (qPCR) was performed using the Step One Plus system (Applied Biosystems, Foster City, CA, USA) according to the SYBR Green (Takara, Dalian, China) protocol, with β-actin as an endogenous control. Primer sequences used in qPCR were found in Table S3. Relative mRNA expression levels were calculated from corresponding relative quantification (RQ) values and normalized to β-actin expression.

**Construction of lentivirus and stable cell lines**

The lentivirus for XIST-silenced were purchased from Obio Technology (Shanghai) Corp.,Ltd and named lv-shXIST-1, lv-shXIST-2 and lv-shXIST-3. The empty pLKD-CMV-G&PR-U6-shRNA lentiviral vector was used as negative control (lv-shControl). To achieve lentiviral infection, HCC-1016 cells were plated at a concentration of 70–80% cells in 6-well plates overnight and were infected with the lentivirus for 24 h according to the manufacturer^，^s protocol. The virus-containing culture medium was replaced with fresh high-glucose DMEM medium supplemented with 10% FBS for 72 h. Then, puromycin-resistant cell clones at a concentration of 8 mg/mL for 3 days. Lentiviral infection efficiency was demonstrated by observing the presence of green fluorescent protein with in the cells using an Olympus-IX71 fluorescence microscope (Olympus) and real-time PCR.

**siRNA construct**

The YY1 siRNAs were purchased from Obio Technology (Shanghai) Corp.,Ltd and named siNC, siYY1-1, siYY1-2 and siYY1-3. HCC-1016 cell transfection was performed with Lipofectamine 3000 (Invitrogen) as described in the manufacturer’s protocol. The sequence of YY1 siRNAs were listed in Table S3.

**CCK8 assay**

The Cell Counting Kit-8 (Beyotime) was assessed to detect Cell proliferation following the manufacturer’s recommendations. Briefly, stable HCC-1016 cells were seeded at a density of 4×10^3^ cells per well in 96-well plates. After adhesion, 10 μL of CCK-8 solution was added to each well, followed by incubation at 37℃ for 2 h. Cell proliferation curves were plotted by measuring the absorbance at 450 nm every 12 h.

**EdU immunofluorescence staining assay**

The EdU (5-ethynyl-2’-deoxyuridine) proliferation assay was performed using the Cell-Light EdU Apollo567 In vitro Kit (Ribobio) according to the manufacturer’s protocol. Images were captured with a Zeiss axiophot photomicroscope (Carl Zeiss) and EdU-positive and total cells were counted in five random fields from each sample.

**Immunohistochemical assay**

immunohistochemistry (IHC) analysis was conducted to determine TET2 (ab94580, 1:200) protein expression in HCC tissues and adjacent normal liver tissues from 30 female patients, and Ki67 (ab16667, 1:200), CD31 (ab182981, 1:200) in mice liver tissues by Servicebio Technology Co.Ltd. We scanned specimens with a Pannoramic Scan (3DHistech, Budapest, Hungary). Visual analysis of digital slides was performed using QuantCenter (3DHistech, Budapest, Hungary). We calculated the number of stained cells in each region of the processed digital sections with the corresponding quantification modules, which had been visually analyzed using QuantCenter (3DHistech, Budapest, Hungary), and reported negative and positive cell counts and their percentages, according to staining intensity (negative, weak, moderate, or strong). Finally calculated the H-score for each region based on the default settings determined by the resulting percentage and intensity of positive cell. At least 2 professional pathologists were invited to score, and the results verified the accuracy of computer-aided measurements.
